# Lipid control in a cohort of patients with cardiorenal disease (LIPID-REN): a subanalysis of the CARDIOREN registry

**DOI:** 10.1093/ckj/sfag369

**Published:** 2026-10-15

**Authors:** Ramón Garrido-González, Almudena Castro, Rafael de la Espriella, Jara Gayán Ordás, Isabel Zegrí-Reiriz, Pau Llácer, Aleix Fort, Adriana Rodríguez Chavarri, Ana Méndez, Zorba Blázquez-Bermejo, Pedro Caravaca Pérez, Jorge Rubio Gracia, Alejandro Recio-Mayoral, Jose Manuel García Pinilla, Carlos Collado Macián, Jose Luis Górriz, Maria Jose Soler, Julio Núñez, Marta Cobo Marcos

**Affiliations:** Department of Cardiology, Hospital Universitario Puerta de Hierro Majadahonda (IDIPHISA), Madrid, Spain; Centro de Investigación Biomédica en Red en Enfermedades Cardiovasculares (CIBERCV), Madrid, Spain; Department of Cardiology, Hospital Universitario La Paz, Madrid, Spain; Department of Cardiology, Hospital Clínico Universitario de Valencia (INCLIVA), Valencia, Spain; Department of Cardiology, Hospital Universitario Arnau de Vilanova, Institut de Recerca Biomèdica de Lleida (IRBLleida), Lleida, Spain; Department of Cardiology, Hospital de la Santa Creu i Sant Pau, Barcelona, Spain; Internal Medicine Department, Hospital Universitario Ramón y Cajal, IRYCIS, Madrid, Spain; Department of Medicine and Medical Specialties, Facultad de Medicina y Ciencias de la Salud, Universidad de Alcalá, Madrid, Spain; Department of Cardiology, Hospital Universitari Dr. Josep Trueta, Girona, Spain; Department of Cardiology, Hospital Universitario La Paz, Madrid, Spain; Department of Cardiology, Hospital Universitario Vall d’Hebron, Barcelona, Spain; Department of Cardiology, Hospital Universitario Gregorio Marañón, Madrid, Spain; Department of Cardiology, Hospital Universitario Doce de Octubre, Madrid, Spain; Department of Internal Medicine, Hospital Universitario Lozano Blesa, University of Zaragoza, Zaragoza, Spain; Department of Cardiology, Hospital Universitario Virgen Macarena, Sevilla, Spain; Department of Cardiology, Hospital Universitario Virgen de la Victoria, Málaga, Spain; Department of Cardiology, Hospital Universitario Puerta de Hierro Majadahonda (IDIPHISA), Madrid, Spain; Department of Nephrology, Hospital Clínico Universitario de Valencia (INCLIVA), University of Valencia, Valencia, Spain; RICORS2040 (RD24/0004/0022), Instituto de Salud Carlos III, Madrid, Spain; Department of Nephrology, Hospital Universitario Vall d’Hebron, Barcelona, Spain; Centro de Investigación Biomédica en Red en Enfermedades Cardiovasculares (CIBERCV), Madrid, Spain; Department of Cardiology, Hospital Clínico Universitario de Valencia (INCLIVA), Valencia, Spain; Department of Cardiology, Hospital Universitario Puerta de Hierro Majadahonda (IDIPHISA), Madrid, Spain; Centro de Investigación Biomédica en Red en Enfermedades Cardiovasculares (CIBERCV), Madrid, Spain

**Keywords:** atherosclerosis, cardiorenal disease, chronic renal failure, dyslipidemia, heart failure

## Abstract

**Aims:**

Advanced chronic kidney disease (CKD) confers a very high cardiovascular disease (CVD) risk, requiring comprehensive risk factor assessment. However, real-world data on current practices in patients with cardiorenal disease remain limited. We aimed to evaluate the control of cardiovascular risk factors, with a focus on lipid management, in a contemporary cardiorenal cohort.

**Methods:**

This cross-sectional analysis was conducted within the CARDIOREN Registry, which enrolled patients with chronic heart failure from 13 tertiary hospitals in Spain (2021–2022). Of 1107 patients, 885 with complete data on low-density lipoprotein cholesterol (LDL-C), albuminuria, and atherosclerotic CVD (ASCVD) were eligible. The present analysis was restricted to the patients who met the definition of advanced CKD, defined as estimated glomerular filtration rate (eGFR) <30 ml/min/1.73 m² or eGFR 30–45 ml/min/1.73 m² with albuminuria >30 mg/g. ASCVD was defined as prior ischemic heart disease, stroke, or peripheral arterial disease. The proportion of patients with LDL-C <55 mg/dl was assessed. Multivariable logistic regression models, including a parsimonious primary model and sensitivity analyses, were used to identify factors associated with lower LDL-C levels.

**Results:**

Advanced CKD was present in 221 patients (25%); median age was 79 years, 58.8% were male, and 52.9% had ASCVD. Median LDL-C was 73 mg/dl, and only 22.6% achieved an LDL-C <55 mg/dl. Achievement of LDL-C <55 mg/dl was significantly lower in patients without ASCVD compared with those with ASCVD (13.4% vs. 30.7%; *P* = .002). In multivariable analysis, ASCVD was independently associated with a higher likelihood of achieving LDL-C <55 mg/dl, whereas female sex was independently associated with a lower likelihood of achieving this level.

**Conclusions:**

In patients with advanced CKD, lipid control was below cardiology guideline recommendations for patients at very high CVD risk, particularly among those without ASCVD and in women.

## Graphical Abstract

**Graphical Abstract:**
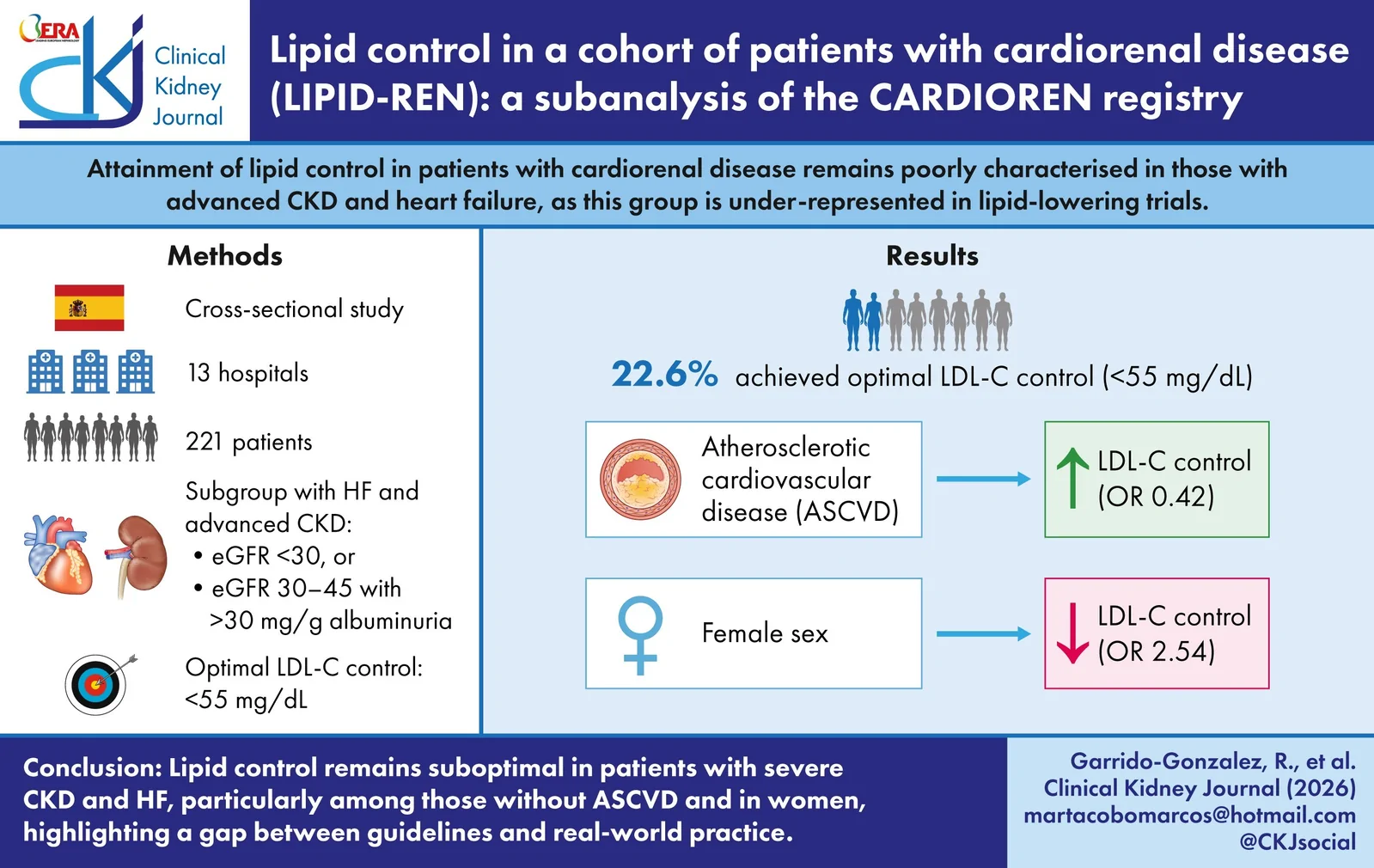

KEY LEARNING POINTS **What was known:**

- Advanced chronic kidney disease (CKD) confers very high cardiovascular risk, and current 2021 ESC Guidelines on cardiovascular disease prevention in clinical practice recommend low-density lipoprotein cholesterol (LDL-C) targets <55 mg/dl regardless of atherosclerotic cardiovascular disease (ASCVD).
- Patients with CKD are underrepresented in lipid-lowering trials, limiting real-world evidence.
- Previous studies have reported a low proportion of patients with LDL-C <55 mg/dl in high-risk populations, but data in cardiorenal disease remain limited.

**This study adds:**

- In a contemporary cardiorenal cohort, fewer than one in four patients achieved LDL-C <55 mg/dl.
- Patients without ASCVD were less likely to have LDL-C <55 mg/dl despite very high risk, suggesting under-recognition of CKD as a risk equivalent.
- Female sex was independently associated with higher odds of LDL-C ≥55 mg/dl, highlighting sex-based disparities.

**Potential impact:**

- These findings describe real-world lipid management in patients with cardiorenal disease.
- Improving recognition of CKD as a cardiovascular risk equivalent may enhance treatment intensification.
- Multidisciplinary strategies and optimized lipid-lowering therapy could reduce residual cardiovascular risk.

## INTRODUCTION

Chronic kidney disease (CKD) markedly amplifies cardiovascular (CV) risk and is recognized as a major CV risk condition in current prevention guidelines [1−3]. Advanced CKD frequently coexists with heart failure (HF), forming a complex cardiorenal phenotype characterized by advanced age, multimorbidity, and a high burden of adverse outcomes [4]. Despite this very high CV risk, patients with advanced CKD have been systematically underrepresented in lipid-lowering trials, resulting in limited real-world evidence on the implementation and effectiveness of contemporary lipid management strategies in this population [ 5, 6].

Importantly, the optimal low-density lipoprotein cholesterol (LDL-C) goal in patients with CKD remains debated. Although 2021 ESC Guidelines on CV disease (CVD) prevention classify advanced CKD as a very high-risk condition warranting an LDL-C target of <55 mg/dl irrespective of established atherosclerotic CVD (ASCVD) [3], this recommendation is largely extrapolated rather than supported by CKD-specific treat-to-target trials [7]. Nevertheless, assessing the achievement of these recommended targets in routine clinical practice remains clinically relevant, particularly among patients with advanced CKD and no documented ASCVD, in whom real-world data are scarce [ 5, 6]. This gap between guideline recommendations and actual LDL-C control may represent a missed opportunity for CV risk recognition in a particularly vulnerable population.

Therefore, we aimed to assess the degree of control of CV risk factors, with a specific focus on LDL-C management, in a contemporary cohort of patients with cardiorenal disease enrolled in the Cardioren Registry, stratified by ASCVD status.

## MATERIALS AND METHODS

### Study design and population

This cross-sectional analysis is a prespecified substudy of the CARDIOREN Spanish Registry, a prospective, multicenter registry that enrolled consecutive ambulatory patients with chronic HF attending specialized HF clinics in 13 tertiary hospitals in Spain between October 2021 and February 2022. The diagnosis of HF was based on clinical assessment by the treating physician, consistent with ESC criteria, irrespective of left ventricular ejection fraction [8]. The primary objective of the CARDIOREN Registry was to assess the prevalence and clinical profile of kidney disease in a contemporary population of patients with chronic HF, in whom kidney disease was highly prevalent, affecting ~70% of the cohort [4]. During the study period, clinical management across participating centers was guided by contemporary European recommendations, including the 2021 ESC Guidelines on CVD prevention in clinical practice [3] and the 2021 ESC Guidelines for the diagnosis and treatment of acute and chronic HF [8].

For the present substudy, focused on CV risk factors, patients with complete information on estimated glomerular filtration rate (eGFR), albuminuria, LDL-C levels, and ASCVD status were included.

Of the 1107 patients enrolled in the CARDIOREN registry, 885 had complete data on eGFR, albuminuria, LDL-C, and ASCVD status. The present analysis was restricted to the 221 patients who met the definition of advanced CKD and constituted the study population.

Advanced CKD was defined as an eGFR <30 ml/min/1.73 m² or an eGFR between 30 and 45 ml/min/1.73 m² in the presence of albuminuria >30 mg/g [3], thereby identifying the subgroup at very high CV risk analyzed in this study. The eGFR was calculated using the CKD-EPI equation [9]. ASCVD was considered present in patients with a documented history of ischemic heart disease, ischemic stroke, or peripheral arterial disease.

Optimal lipid control was defined as LDL-C <55 mg/dl, in accordance with contemporary European Prevention Guidelines [3]. High-intensity statin therapy was defined as treatment with atorvastatin ≥40 mg/day or rosuvastatin ≥20 mg/day. Adequate blood pressure control was defined as systolic/diastolic blood pressure <140/90 mmHg, consistent with the general target recommended in the 2021 ESC Prevention Guidelines for patients at high CV risk, and adequate glycemic control as glycated hemoglobin ≤7.0% in the presence of diabetes mellitus [3]. Body mass index (BMI) was calculated as weight divided by height squared (kg/m²) and categorized according to World Health Organization criteria as normal weight (18.5–24.9 kg/m²), overweight (25.0–29.9 kg/m²), and obesity (≥30.0 kg/m²).

### Data collection

Baseline data included demographics, CV risk factors, comorbidities, clinical status, laboratory parameters, and background pharmacological therapies. Laboratory analyses, including LDL cholesterol, N-terminal pro-B-type natriuretic peptide (NT-proBNP), hemoglobin, serum creatinine, and urine albumin-to-creatinine ratio, were obtained at the baseline visit and analyzed in local laboratories.

### Statistical analysis

Continuous variables are reported as median (interquartile range), and categorical variables as frequencies and percentages. Comparisons between groups were performed using the *χ*² test for categorical variables and appropriate parametric or nonparametric tests for continuous variables. Factors independently associated with not achieving LDL-C <55 mg/dl (LDL-C ≥55 mg/dl) were examined by multivariable logistic regression. Because model stability in logistic regression is governed by the minority outcome class, the relevant count was the 50 patients achieving the target rather than the 171 above it; we therefore prespecified a parsimonious model including the four covariates of greatest clinical relevance and biological plausibility (ASCVD status, sex, age, and diabetes mellitus), corresponding to an events-per-variable ratio of 12.5. To limit small-sample bias, this model was additionally refitted using Firth’s penalized-likelihood method, and univariable odds ratios (ORs) are reported for all covariates. Continuous covariates were modeled linearly after checking for departures from linearity. Lipid-lowering therapy, which lies on the causal pathway to LDL-C, was analyzed primarily descriptively to avoid mediation/collider bias. As sensitivity analyses, fuller models additionally incorporating NT-proBNP, hemoglobin, and lipid-lowering therapy were fitted to assess the robustness of the main associations. Results are presented as ORs with 95% confidence intervals (CIs). Statistical significance was set at a two-sided *P*-value <.05.

As an additional analysis, we evaluated factors associated with the prescription of high-intensity statin therapy, defined as atorvastatin ≥40 mg/day or rosuvastatin ≥20 mg/day, compared with lower-intensity or no statin therapy. This model included age, sex, diabetes mellitus, ASCVD status, NT-proBNP, and hemoglobin.

Finally, to assess LDL-C target attainment across the spectrum of renal function, we compared the proportion of patients achieving LDL-C targets across eGFR categories in the overall cohort (*n* = 885).

Analyses were performed using Stata software (StataCorp 16.1 StataCorp LLC).

## RESULTS

### Baseline characteristics

Among the 885 patients eligible in this subanalysis, 221 (25.0%) met criteria for advanced CKD and constituted the population of this substudy. Of these, 123 patients (55.7%) had an eGFR <30 ml/min/1.73 m², while 98 (44.3%) had an eGFR between 30 and 45 ml/min/1.73 m² with albuminuria. The median age was 79 years (IQR 73–85), 58.8% were male, and 52.9% had established ASCVD. Regarding HF phenotype, 92 patients (41.6%) had HFrEF, 32 (14.5%) had HFmrEF, and 97 (43.9%) had HFpEF (Table 1). Patients with advanced CKD were older, had poorer functional status, and carried a substantial burden of comorbidities, along with markedly elevated NT-proBNP and CA125 levels, reflecting a more advanced cardiorenal phenotype (Supplementary Material 1).

**Table 1:** Baseline characteristics in patients with advanced CKD stratified by the presence of ASCVD.

Variable	Total (*n* = 221)	No ASCVD (*n* = 104, 47.1%)	ASCVD (*n* = 117, 52.9%)	*P* value
Age (years)	79 (73, 85)	79.5 (74.5, 85.5)	79.0 (70.0, 83.0)	.065
Sex (male), *n* (%)	130 (59)	49 (47)	81 (69)	.001
Smoking, *n* (%)	111 (50)	44 (42)	67 (57)	.026
Active	13 (6)	7 (6)	6 (6)	
Former	98 (44)	38 (36)	60 (51)	
Diabetes, *n* (%)	137 (62)	57 (55)	80 (68)	.038
HT, *n* (%)	197 (89)	91 (88)	106 (91)	<.001
COPD, *n* (%)	42 (19)	19 (18)	23 (20)	.793
AF, *n* (%)	140 (63)	72 (69)	68 (58)	.087
NYHA class, *n* (%)				.557
I	14 (6.4)	5 (4.9)	9 (7.8)	
II	144 (65.8)	67 (65.0)	77 (66.4)	
III	60 (27.4)	30 (29.1)	30 (25.9)	
IV	1 (0.5)	1 (1.0)	0 (0.0)	
Ischemic heart disease, *n* (%)	86 (39)	/	86 (74)	
Stroke, *n* (%)	37 (17)	/	37 (32)	
Peripheral arterial disease, *n* (%)	34 (15)	/	34 (29)	
SBP (mmHg)	125 (110, 142)	125 (110 143)	125 (110 140)	.956
DBP (mmHg)	70 (62,76)	69 (60,75)	70 (63,77)	.214
BMI (kg/m^2^)	28 (25,32)	28.1 (24.8,31.2)	27.9 (24.6,32.2)	.865
BMI categories Underweight Normal Overweight Obesity	2 (0.9) 56 (25) 79 (36) 84 (38)	1 (0.9) 26 (25) 39 (37) 38 (37)	1 (0.9) 30 (26) 40 (34) 46 (39)	.961
Hemoglobin (g/dl)	12.3 (11.2,13.8)	12.3 (11.3,13.4)	12.3 (11.2,14.2)	.550
Creatinine (mg/dl)	1.9 (1.6,2.3)	1.8 (1.6,2.2)	1.9 (1.7,2.4)	.040
UACR (mg/g)	80 (40 230)	70 (35 195)	90 (40 250)	.208
HbA1c (%)	6.1 (5.6,6.8)	5.9 (5.5,6.4)	6.1 (5.8,7.2)	.004
TG (mg/dl)	109 (84 152)	111 (82 145)	108 (87 159)	.524
Cholesterol (mg/dl)	142 (118 170)	154 (132 189)	133 (115, 156)	<.001
LDL (mg/dl)	73 (57 100)	91 (64 118)	65 (52,84)	<.001
HDL (mg/dl)	43 (36,54)	47 (39,57)	42 (35,52)	.013
NTproBNP (pg/ml)	3547 (1593,7590)	3748 (1720,7590)	3304 (1563,7529)	.944
CA125 (U/l)	24 (13,64)	29 (14,75)	21 (11,44)	.059
LVEF (%)	45 (33, 58)	54 (41,61)	37 (30,55)	<.001
HFrEF HFmrEF HFpEF	92 (42) 32 (14) 97 (44)	26 (28) 16 (50) 62 (64)	66 (72) 16 (50) 35 (36)	<.001
Furosemide (mg)	80 (40 120)	20 (0,60)	80 (40 120)	<.001
SGLT2i, *n* (%)	116 (53)	46 (44)	70 (60)	.020
Beta-blockers, *n* (%)	163 (74)	73 (70)	90 (77)	.256
RASI, *n* (%)	132 (60)	58 (56)	74 (63)	.258
MRA, *n* (%)	68 (35)	37 (36)	41 (35)	.934
Statin, *n* (%)	153 (69)	59 (57)	94 (80)	<.001
Ezetimibe, *n* (%)	30 (14)	7 (7)	23 (20)	.006
PCSK9i, *n* (%)	1 (0.5)	0 (0)	1 (1)	.349
Combined therapy, *n* (%)	30 (14)	7 (7)	23 (20)	.005
GLP1a, *n* (%)	20 (9)	8 (8)	12 (10)	.507

Abbreviations: CKD, chronic kidney disease; HT, hypertension; COPD, chronic obstructive pulmonary disease; AF, atrial fibrillation; NYHA, New York Heart Association functional class; SBP, systolic blood pressure; DBP, diastolic blood pressure; BMI, body mass index; UACR, urinary albumin-to-creatinine ratio; HbA1c, glycated hemoglobin; TG, triglycerides; LDL, low-density lipoprotein cholesterol; HDL, high-density lipoprotein cholesterol; NT-proBNP, N-terminal pro-B-type natriuretic peptide; CA125, cancer antigen 125; LVEF, left ventricular ejection fraction; HFrEF, heart failure with reduced ejection fraction; HFmrEF, heart failure with mildly reduced ejection fraction; HFpEF, heart failure with preserved ejection fraction; SGLT2i, sodium–glucose co-transporter 2 inhibitors; RASI, renin–angiotensin system inhibitors; MRA, mineralocorticoid receptor antagonists; PCSK9i, proprotein convertase subtilisin/kexin type 9 inhibitors; GLP-1a, glucagon-like peptide-1 receptor agonists.

Baseline characteristics according to ASCVD status in the population with advanced CKD are shown in Table 1. Patients with ASCVD were more frequently male and had a higher prevalence of diabetes mellitus, hypertension, and smoking history, as well as a lower left ventricular ejection fraction. Lipid profiles differed by ASCVD status, with lower total cholesterol and LDL-C levels among patients with ASCVD. The use of lipid-lowering therapies was higher in patients with ASCVD, including statins and ezetimibe, whereas PCSK9 inhibitors remained low. Patients with ASCVD were also more frequently treated with SGLT2 inhibitors, whereas the use of other guideline-directed HF therapies was comparable between groups.

### Cardiovascular risk factors control

The blood pressure threshold <140/90 was observed in 70% of patients with advanced CKD, with no significant difference between those with (70%) and without (69%) ASCVD (*P* = .890).

Among patients with diabetes, inadequate glycemic control was observed in ~35% of the cohort, affecting 27.4% of patients without ASCVD and 39.7% of those with ASCVD, with no statistically significant difference between groups (*P* = .152).

Overall, 75% of patients had a BMI ≥25 kg/m², including 38% who met criteria for obesity. The median BMI was 27.9 kg/m² (IQR 24.6–31.6), with BMI distributions comparable across ASCVD status (Table 1).

Median LDL cholesterol was 73 mg/dL (IQR 57–100). Only 50 patients (22.6%) achieved an LDL-C <55 mg/dl. Achievement of LDL-C <55 mg/dl was significantly lower in patients without ASCVD than in those with established ASCVD (13.4% vs. 30.7%; *P* = .002). This disparity remained evident even when less stringent LDL-C thresholds were applied. LDL-C levels <70 mg/dl were achieved in 45.7% of patients, with substantially lower attainment among those without ASCVD compared with those with established ASCVD (30.7% vs. 58.9%; *P* < .001). Similarly, LDL-C <100 mg/dl was achieved in 63% of patients without ASCVD, compared with 87.1% of those with ASCVD (*P* < .001) (Fig. 1).

**Figure 1:**
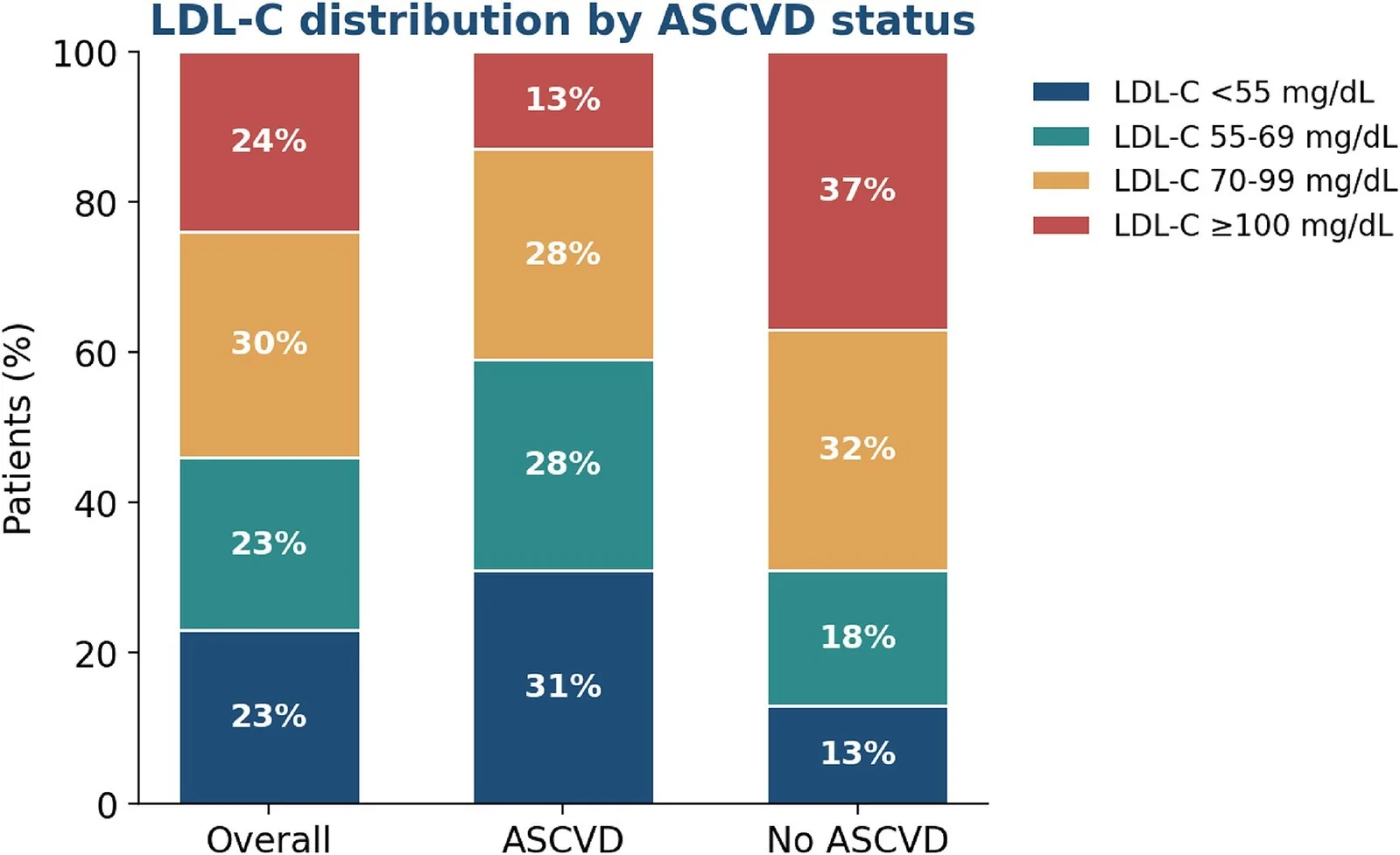
LDL-C control stratified by the presence of atherosclerotic CVD in advanced kidney disease. LDL-c, low-density lipoprotein cholesterol; ASCVD, atherosclerotic cardiovascular disease.

### LDL-C control across the renal-function spectrum

To place LDL-C target attainment in the context of renal function, we compared achievement of LDL-C goals across eGFR categories in the overall eligible cohort (*n* = 885). LDL-C <55 mg/dl attainment differed across eGFR strata but did not follow a graded pattern with declining renal function (eGFR ≥ 60: 16.4%; 45–60: 28.3%; 30–45: 17.0%; <30: 24.4%; *P* = .004 across categories). Accordingly, patients with advanced CKD did not show higher LDL-C <55 mg/dl attainment than the rest of the cohort (22.6% vs. 19.1%; *P* = .30). Similar findings were observed when LDL-C thresholds of <70 and <100 mg/dl were considered (Supplementary Material 2).

### Lipid-lowering therapies

In the overall cohort of patients with advanced CKD, 69% of patients were on statins, 14% on ezetimibe, and 14% on both. Among the 153 patients receiving statins, high-intensity statin therapy was prescribed in 51%. The distribution of mutually exclusive lipid-lowering regimens according to ASCVD status is shown in Fig. 2. Patients without ASCVD were more frequently untreated than those with ASCVD (43% vs. 20%). Conversely, patients with ASCVD more frequently received high-intensity statin monotherapy (35% vs. 12%) and statin plus ezetimibe combination therapy (20% vs. 7%), whereas low- or moderate-intensity statin monotherapy was more frequent among patients without ASCVD (38% vs. 26%).

**Figure 2:**
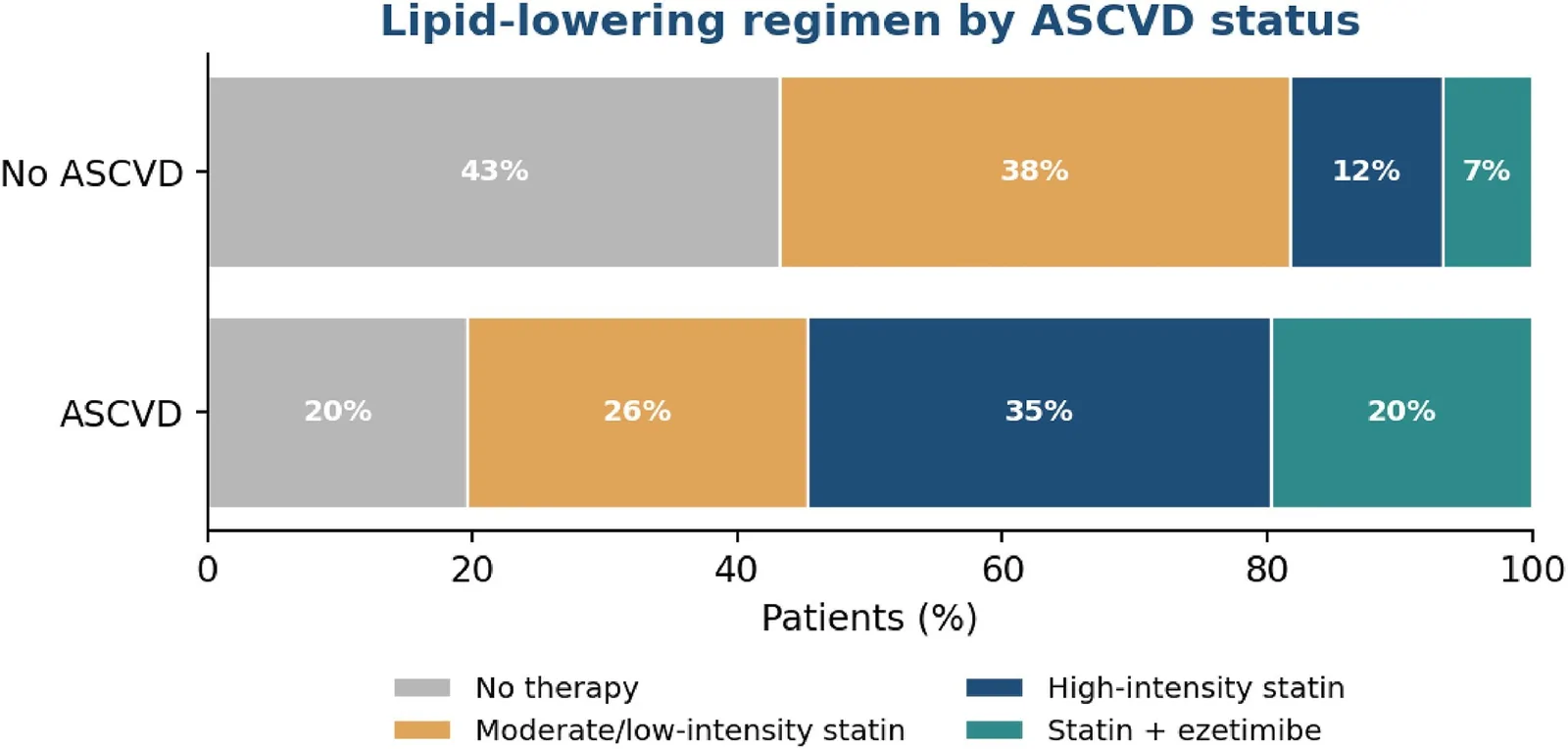
Lipid-lowering regimen by ASCVD status in advanced kidney disease. Stacked bar chart comparing lipid-lowering treatments by ASCVD status, showing more frequent use of high-intensity statins and statin–ezetimibe combination therapy in patients with ASCVD.

LDL-C target attainment increased progressively with treatment intensity, from 11.8% in untreated patients to 25.7%, 26.4%, and 33.3% with moderate/low-intensity statins, high-intensity statins, and statin–ezetimibe combination, respectively (Supplementary Material 3), and was consistently higher in patients with ASCVD at every regimen.

In an additional multivariable model evaluating factors associated with high-intensity statin therapy versus lower-intensity or no statin therapy, established ASCVD was the only variable independently associated with high-intensity statin prescription. Patients with ASCVD had more than six-fold higher odds of receiving high-intensity statins than those without ASCVD (OR 6.37, 95% CI 2.62–15.50; *P* < .001).

### Factors associated with LDL-C control

In the parsimonious multivariable model, two factors were independently associated with not achieving LDL-C <55 mg/dl. Established ASCVD was associated with lower odds of not achieving LDL-C <55 mg/dl (OR 0.44; 95% CI 0.21–0.89; *P* = .023), whereas female sex was associated with higher odds of not achieving LDL-C <55 mg/dl (OR 2.62; 95% CI 1.22–5.63; *P* = .013; Table 2). Estimates were essentially unchanged with Firth penalization (ASCVD OR 0.45, 95% CI 0.22–0.91; female sex OR 2.52, 95% CI 1.19–5.33) and were consistent with the univariable associations (ASCVD OR 0.35, 95% CI 0.18–0.70; female sex OR 3.12, 95% CI 1.50–6.49), confirming robustness despite the limited number of events (events-per-variable 12.5).

**Table 2:** Multivariable logistic regression models for not achieving LDL-C <55 mg/dl.

Variable	OR (95% CI)	*P*
Primary model—parsimonious (Firth-penalized)		
ASCVD (vs. none)	0.45 (0.22–0.91)	.026
Female sex (vs. male)	2.52 (1.19–5.33)	.016
Age (per year)	1.00 (0.97–1.04)	.847
Diabetes mellitus	0.72 (0.36–1.44)	.355
Sensitivity model 1—fully adjusted		
ASCVD (vs. none)	0.42 (0.18–0.98)	.045
Female sex (vs. male)	2.54 (1.06–6.12)	.038
Age (per year)	0.99 (0.94–1.03)	.541
NT-proBNP (per 1000 pg/ml)	0.95 (0.91–1.00)	.075
Hemoglobin (per g/dl)	1.18 (0.97–1.44)	.090
Diabetes mellitus	0.72 (0.31–1.66)	.442
Statin therapy	0.49 (0.17–1.37)	.172
Ezetimibe therapy	0.62 (0.23–1.67)	.341
Sensitivity model 2—excluding lipid-lowering therapy		
ASCVD (vs. none)	0.40 (0.18–0.88)	.024
Female sex (vs. male)	2.71 (1.14–6.44)	.024
Age (per year)	1.00 (0.95–1.04)	.834
NT-proBNP (per 1000 pg/ml)	0.95 (0.90–1.00)	.045
Hemoglobin (per g/dl)	1.16 (0.96–1.39)	.121
Diabetes mellitus	0.65 (0.30–1.43)	.285

Note: OR > 1 indicates higher odds of not achieving LDL-C <55 mg/dl (LDL-C ≥55 mg/dl). The primary model (four covariates; events-per-variable 12.5, based on the 50 patients at target) is shown with Firth penalization; the standard adjusted estimates were essentially identical (ASCVD 0.44, 95% CI 0.21–0.89; female sex 2.62, 95% CI 1.22–5.63). Lipid-lowering therapy was analyzed descriptively (mediator). Values are odds ratios with 95% confidence intervals. NT-proBNP was entered per 1000 units.

Abbreviations: ASCVD, atherosclerotic cardiovascular disease; CI, confidence interval; LDL-C, low-density lipoprotein cholesterol; NT-proBNP, N-terminal pro-B-type natriuretic peptide; OR, odds ratio.

In sensitivity analyses additionally adjusting for NT-proBNP and hemoglobin, and in a further model incorporating lipid-lowering therapy, the associations of ASCVD and female sex with LDL-C control were unchanged (ASCVD OR 0.40–0.42; female sex OR 2.54–2.71; all *P* < .05). In these models, higher NT-proBNP was modestly associated with lower odds of not achieving LDL-C <55 mg/dl (OR 0.95 per 1000 pg/ml increase; 95% CI 0.90–1.00; *P* = .045).

In a sex-stratified analysis, women showed lower use of intensive lipid-lowering strategies and lower LDL-C target attainment compared with men. The use of any statin, high-intensity statin therapy, ezetimibe, and combination therapy, as well as LDL-C <55 mg/dl achievement, according to sex, are shown in Supplementary Material 4.

## DISCUSSION

This contemporary real-world cohort of patients with advanced cardiorenal disease reveals several clinically relevant findings. First, in patients with advanced CKD and HF, LDL-C levels were below cardiology guideline recommendations for patients at very high CV risk [3], with fewer than one in four patients achieving LDL-C <55 mg/dl. Second, patients with advanced CKD but without established ASCVD, despite being at very high CV risk, exhibited significantly lower achievement of LDL-C <55 mg/dl than their counterparts with documented ASCVD, underscoring a persistent perception gap regarding the CV risk associated with advanced CKD. Third, female sex emerged as an independent determinant of a lower likelihood of achieving LDL-C <55 mg/dl, highlighting a relevant sex-based disparity in CV risk management within this high-risk population.

### Advanced CKD as a risk equivalent, with caveats about the target

Our findings should be interpreted in the context of the ongoing uncertainty regarding the optimal LDL-C goal in patients with CKD. Although European guidelines classify patients with advanced CKD as at high or very high CV risk and recommend intensive lipid lowering according to their estimated risk category [3], the specific LDL-C target of <55 mg/dl in very high-risk CKD is largely extrapolated from the broader CV prevention framework rather than established by CKD-specific treat-to-target trials. Indeed, the pivotal SHARP trial [7] demonstrated that simvastatin–ezetimibe reduces major atherosclerotic events in CKD, but it did not evaluate LDL-C targets, and evidence supporting high-intensity lipid-lowering regimens in advanced CKD remains limited, particularly in patients receiving kidney replacement therapy.

Nevertheless, patients with advanced CKD represent a population with a markedly increased risk of CV events, even in the absence of documented ASCVD. This high residual risk provides the clinical rationale for the risk stratification and LDL-C goals proposed by the European Society of Cardiology. Accordingly, we do not interpret low LDL-C target attainment as failure to meet a definitively proven CKD-specific therapeutic mandate, but rather as a signal of potential under-recognition and undertreatment of CV risk in this vulnerable population.

Importantly, the pattern observed in our study was consistent across several LDL-C thresholds and was largely driven by the presence or absence of documented ASCVD. Patients without ASCVD, in whom treatment decisions rely more heavily on CKD-related CV risk, showed the lowest likelihood of achieving recommended LDL-C levels, and target attainment did not improve as renal function declined. These findings suggest that renal risk alone may be insufficiently recognized in routine clinical practice.

Our additional analysis assessing treatment intensity showed that high-intensity statin therapy was mainly driven by ASCVD status. However, further studies including treatment duration, adherence, intolerance, and longitudinal LDL-C trajectories are needed to clarify whether low target attainment reflects therapeutic inertia, clinical caution, or patient-related factors.

In addition, low LDL-C levels in untreated patients should be interpreted carefully, as they may reflect frailty, malnutrition, dietary restriction, or other non-treatment-related factors rather than optimal CV prevention.

### Comparison with previous very high-risk/CKD cohorts

Low LDL-C target attainment has been consistently reported in registries of patients at very high CV risk. In the Santorini registry, only 20% of very high-risk patients achieved LDL-C levels <55 mg/dl, and EUROASPIRE surveys have shown similar rates of 20%–30% in secondary prevention settings [ 10, 11]. However, few studies have specifically assessed lipid management in the cardiorenal population. Our results are consistent with the CKD-REIN cohort, in which LDL-C <70 mg/dl was achieved in only 29% of patients with CKD stages G4–G5, compared with 46% in our cohort [5]. Importantly, our study adds insight by stratifying patients according to ASCVD status, revealing that the presence of overt ASCVD strongly influences target achievement even within a population uniformly classified as very high risk on the basis of renal function alone.

### Lipid-lowering therapies: intensity and combination therapy

Although lipid-lowering therapy was more frequent among patients with ASCVD, overall treatment intensity remained modest. Only half of statin-treated patients received high-intensity statins, ezetimibe use was low, and PCSK9 inhibitors were virtually absent. These findings mirror those of other European registries [10]. The therapeutic landscape during the study period should be considered: bempedoic acid and inclisiran were not yet available in routine clinical practice, and PCSK9 inhibitor access was subject to strict reimbursement criteria, likely contributing to the low uptake of advanced lipid-lowering therapies.

### Determinants of LDL-C control: insights from multivariable analysis

The presence of established ASCVD emerged as the strongest independent predictor of achieving LDL-C targets, reinforcing that lipid management in advanced CKD remains driven by secondary prevention paradigms. Female sex was independently associated with a lower likelihood of achieving LDL-C <55 mg/dl, consistent with previously described sex-based disparities in CV prevention [10], and may reflect differences in risk perception, treatment intensification, or tolerability concerns in older women.

Interestingly, although ASCVD was strongly associated with high-intensity statin prescription, adjustment for lipid-lowering therapy only minimally changed the association between ASCVD and not achieving LDL-C <55 mg/dl. These findings should be interpreted as complementary rather than contradictory. ASCVD may act not only as a determinant of treatment intensity at the baseline visit, but also as a broader marker of a secondary-prevention care pathway, including greater risk perception, longer exposure to lipid-lowering therapy, closer follow-up, more frequent treatment intensification over time, and potentially better adherence.

### Beyond lipid management: control of other cardiovascular risk factors

Beyond lipid management, blood pressure target attainment was observed in ~70% of patients, consistent with contemporary CKD and HF registries [ 12, 13]. Glycemic control among patients with diabetes was suboptimal in ~35%, comparable to other cardiorenal cohorts [ 14, 15]. Excess body weight was highly prevalent, with three-quarters of patients being overweight or obese [ 16, 17]. Notably, no assessment of visceral adiposity was performed, reflecting limited awareness in routine practice that visceral fat represents an independent CV risk factor. Taken together, these findings reinforce that comprehensive CV risk factor management remains challenging in advanced cardiorenal disease.

### Clinical implications and future directions

Our findings have several important clinical implications. First, although low LDL-C target attainment has been documented in various very high-risk populations, few studies have specifically examined lipid management in patients with the combined cardiorenal phenotype of HF and advanced CKD, stratified by ASCVD status. Our data highlight persistent real-world gaps in preventive care in this particularly vulnerable population. These results reinforce the need to increase clinicians’ awareness that advanced CKD, particularly when accompanied by albuminuria, constitutes a CV risk equivalent, warranting intensive preventive strategies irrespective of the presence of established ASCVD.

Second, our data underscore the importance of earlier and more aggressive lipid-lowering approaches, including the systematic use of high-intensity statins and timely escalation to combination therapy when LDL-C targets are not achieved. In this context, improving the implementation of guideline-recommended therapies within multidisciplinary cardiorenal care pathways, integrating both renal and CV risk markers such as eGFR and albuminuria, should be prioritized rather than relying predominantly on secondary prevention paradigms.

Finally, the identification of female sex as an independent determinant of a lower likelihood of achieving LDL-C <55 mg/dl has relevant clinical implications. This finding highlights the need for greater attention to potential sex-based disparities in CV prevention, ensuring that women with advanced CKD and albuminuria are appropriately risk-stratified and receive equitable access to intensive lipid-lowering strategies. Addressing these gaps may help reduce residual CV risk in a population already characterized by high clinical complexity and therapeutic inertia.

## LIMITATIONS

Several limitations should be acknowledged. First, the cross-sectional design precludes causal inference and does not allow assessment of longitudinal changes in lipid management or clinical outcomes. Second, LDL-C measurements and lipid-lowering treatment data were obtained at a single time point, and information on treatment adherence, prior treatment duration, treatment discontinuation, or statin intolerance was not available. Therefore, we could not determine whether the absence or low intensity of lipid-lowering therapy reflected clinical inertia, physician concerns, patient refusal, poor adherence, or previous discontinuation due to intolerance. Third, lipid assessment in this study was focused on LDL-C, in accordance with current guideline recommendations. However, no data were available on apolipoprotein B, lipoprotein(a), or other markers of atherogenic dyslipidemia. This represents an important limitation, particularly in a population characterized by insulin resistance, obesity, and diabetes, in whom non-LDL-related lipid abnormalities are highly prevalent. These findings also highlight a persistent LDL-centric approach to lipid management in routine clinical practice. Fourth, although the registry reflects real-world practice in specialized HF clinics, the results may not be fully generalizable to other clinical settings. Recruitment from tertiary-center HF clinics likely introduces a selection bias toward more closely monitored patients; consequently, lipid target attainment in the broader CKD population managed in primary care or general cardiology settings may be even lower than reported here. In addition, our study population consists of HF patients with concomitant advanced CKD, and findings should not be extrapolated to CKD populations without HF. Fifth, LDL-C values were obtained from routine laboratory assessments, and fasting status was not systematically recorded; however, current European guidelines do not mandate fasting samples for LDL-C measurement. Sixth, the relatively small number of patients achieving the LDL-C target limits the statistical power of the multivariable model and warrants caution in interpreting null associations, and residual confounding cannot be excluded despite multivariable adjustment. Finally, the lack of longitudinal follow-up data precludes assessment of whether higher LDL-C levels were associated with subsequent CV events, HF hospitalizations, kidney outcomes, or mortality. Therefore, our findings should be interpreted as describing real-world lipid management gaps rather than their prognostic consequences.

## CONCLUSIONS

In this contemporary cohort of patients with cardiorenal disease, LDL-C levels were below cardiology guideline recommendations for patients at very high CV risk, particularly among patients with advanced CKD without established ASCVD and in women. Despite guideline recommendations, lipid-lowering strategies appear to be driven primarily by secondary prevention paradigms rather than renal risk alone. Greater awareness of advanced CKD as a CV risk equivalent and more consistent implementation of intensive lipid-lowering therapies are needed to reduce residual CV risk in this vulnerable population.

## Supplementary Material

sfag369_Supplemental_File

## Data Availability

The data are available on request.
