# Supplementary material for: Lipid control in a cohort of patients with cardiorenal disease (LIPID-REN): a subanalysis of the CARDIOREN registry
Source: Clin Kidney J. 2026 Oct 15;19(11):sfag369. doi: 10.1093/ckj/sfag369 (PMC13748964; doi:10.1093/ckj/sfag369)
Supplement: Supplementary file 1 [file sfag369_supplemental_file.docx]

**Supplementary Table 1. Baseline characteristics in CARDIOREN REGISTRY stratified by the presence of Severe CKD**

| **Variable** | **Total (n=885)** | **No Severe CKD (n=664, 75%)** | **Severe CKD**  **(n=221,25%)** | ***P* value** |
| --- | --- | --- | --- | --- |
| Age (years) | 74 (63,81) | 72 (60,80) | 79 (73,85) | <0.001 |
| Sex (male), n (%) | 565 (64) | 435 (65) | 130 (59) | 0.073 |
| Smoking, n (%) | 448 (51) | 337 (51) | 111 (50) | <0.001 |
| Diabetes, n (%) | 360 (41) | 223 (34) | 137 (62) | <0.001 |
| HT, n (%) | 615 (70) | 418 (63) | 197 (89) | <0.001 |
| COPD, n (%) | 160 (18) | 118 (18) | 42 (19) | 0.680 |
| AF, n (%) | 472 (54) | 332 (50) | 140 (63) | 0.001 |
| NYHA class  n (%) | 130 (14.7)  590 (66.9)  160 (18.1)  2 (0.2) | 116 (17.5)  446 (67.3)  100 (15.1)  1 (0.2) | 14 (6.4)  144 (65.8)  60 (27.4)  1 (0.5) | <0.001 |
| Ischemic Heart Disease, n (%) | 346 (39) | 260 (39) | 86 (39) | 0.949 |
| Ictus, n (%) | 134 (15) | 97 (15) | 37 (17) | 0.443 |
| Peripheral arterial disease, n (%) | 78 (9) | 44 (7) | 34 (15) | <0.001 |
| SBP (mmHg) | 120 (108,135) | 120 (106,131) | 125 (110,142) | <0.001 |
| DBP (mmHg) | 70 (62,77) | 70 (62,77) | 70 (62,76) | 0.593 |
| BMI (kg/m2) | 27.2 (24.4,31.0) | 27.0 (24.3,30.5) | 27.9 (24.7,31.7) | 0.079 |
| Hemoglobin (g/dL) | 13.7 (12.3, 15.0) | 14.1 (12.9,15.4) | 12.3 (11.2,13.8) | <0.001 |
| Creatinine (mg/dL) | 1.2 (0.9, 1.6) | 1.1 (0.8,1.3) | 1.9 (1.6,2.3) | <0.001 |
| UACR (mg/g) | 20 (10,70) | 20 (10,40) | 80 (40,230) | <0.001 |
| HbA1c (%) | 6.0 (5.5,6.5) | 5.9 (5.5,6.4) | 6.1 (5.6,6.8) | 0.006 |
| TG (mg/dL) | 110 (81,150) | 110 (80,150) | 109 (84,152) | 0.437 |
| Cholesterol (mg/dL) | 148 (124,178) | 149 (126,180) | 142 (118, 170) | 0.012 |
| LDL (mg/dL) | 78 (59, 105) | 80 (60,106) | 73 (57,100) | 0.074 |
| HDL (mg/dL) | 45 (38, 54) | 45 (38,54) | 43 (36,54) | 0.049 |
| NTproBNP (pg/mL) | 1369 (577, 3095) | 996 (455,2216) | 3547 (1593,7590) | <0.001 |
| CA125 (U/L) | 15 (9,28) | 13 (9,22) | 24 (13,64) | <0.001 |
| LVEF (%) | 40 (30,55) | 39 (30,53) | 45 (33,58) | <0.001 |
| Furosemide (mg) | 40 (0, 80) | 20 (0,60) | 80 (40,120) | <0.001 |
| SGLT2i, n (%) | 518 (59) | 402 (61) | 116 (53) | 0.035 |
| Betablockers, n (%) | 702 (79) | 539 (81) | 163 (74) | 0.018 |
| RASI, n (%) | 688 (78) | 556 (84) | 132 (60) | <0.001 |
| MRA, n (%) | 517 (58) | 439 (66) | 78 (35) | <0.001 |
| Statin, n (%) | 603 (68) | 450 (68) | 153 (69) | 0.687 |
| Ezetimibe, n (%) | 157 (18) | 127 (19) | 30 (14) | 0.009 |
| PCSK9i, n (%) | 6 (0.7) | 5 (0.8) | 1 (0.5) | 0.635 |
| GLP1a, n (%) | 43 (4.9) | 23 (4) | 20 (9) | 0.001 |

Table Legend: CKD: chronic kidney disease; HT: hypertension; COPD: chronic obstructive pulmonary disease; AF: atrial fibrillation; NYHA: New York Heart Association functional class; SBP: systolic blood pressure; DBP: diastolic blood pressure; BMI: body mass index; UACR: urinary albumin-to-creatinine ratio; HbA1c: glycated hemoglobin; TG: triglycerides; LDL: low-density lipoprotein cholesterol; HDL: high-density lipoprotein cholesterol; NT-proBNP: N-terminal pro–B-type natriuretic peptide; CA125: cancer antigen 125; LVEF: left ventricular ejection fraction; SGLT2i: sodium–glucose cotransporter 2 inhibitors; RASI: renin–angiotensin system inhibitors; MRA: mineralocorticoid receptor antagonists; PCSK9i: proprotein convertase subtilisin/kexin type 9 inhibitors; GLP-1a: glucagon-like peptide-1 receptor agonists.
